# Pulmonary vascular reactivity to supplemental oxygen in Sherpa and lowlanders during gradual ascent to high altitude

**DOI:** 10.1113/EP090458

**Published:** 2022-11-20

**Authors:** Prajan Subedi, Christopher Gasho, Michael Stembridge, Alexandra M. Williams, Alexander Patrician, Philip N. Ainslie, James D. Anholm

**Affiliations:** ^1^ Division of Pulmonary Critical Care, Sleep, Hyperbaric Medicine and Allergy Dept. of Medicine Loma Linda University School of Medicine Pulmonary Section VA Loma Linda Healthcare System Loma Linda California USA; ^2^ Cardiff School of Sport and Health Sciences Cardiff Metropolitan University Cardiff UK; ^3^ Centre for Heart, Lung and Vascular Health Faculty of Health and Social Development University of British Columbia – Okanagan Kelowna BC Canada; ^4^ Department of Cellular and Physiological Sciences Faculty of Medicine University of British Columbia Vancouver BC Canada

**Keywords:** high altitude, hypoxia, hypoxic pulmonary vascular remodelling, hypoxic pulmonary vasoconstriction, Sherpa

## Abstract

Prolonged alveolar hypoxia leads to pulmonary vascular remodelling. We examined the time course at altitude, over which hypoxic pulmonary vasoconstriction goes from being acutely reversible to potentially irreversible. Study subjects were lowlanders (*n* = 20) and two Sherpa groups. All Sherpa were born and raised at altitude. One group (ascent Sherpa, *n* = 11) left altitude and after de‐acclimatization in Kathmandu for ∼7 days re‐ascended with the lowlanders over 8–10 days to 5050 m. The second Sherpa group (non‐ascent Sherpa, *n* = 12) remained continuously at altitude. Pulmonary artery systolic pressure (PASP) and pulmonary vascular resistance (PVR) were measured while breathing ambient air and following supplemental oxygen. During ascent PASP and PVR increased in lowlanders and ascent Sherpa; however, with supplemental oxygen, lowlanders had significantly greater decrease in PASP (*P* = 0.02) and PVR (*P* = 0.02). After ∼14 days at 5050 m, PASP decreased with supplemental oxygen (mean decrease: 3.9 mmHg, 95% CI 2.1–5.7 mmHg, *P* < 0.001); however, PVR was unchanged (*P* = 0.49). In conclusion, PASP and PVR increased with gradual ascent to altitude and decreased via oxygen supplementation in both lowlanders and ascent Sherpa. Following ∼14 days at 5050 m altitude, there was no change in PVR to hypoxia or O_2_ supplementation in lowlanders or either Sherpa group. These data show that both duration of exposure and residential altitude influence the pulmonary vascular responses to hypoxia.

## Abstract (highlights)

- **What is the central question of this study?**
  How does hypoxic pulmonary vasoconstriction and the response to supplemental oxygen change over time at high altitude?
- **What is the main finding and its importance?**
  Lowlanders and partially de‐acclimatized Sherpa both demonstrated pulmonary vascular responsiveness to supplemental oxygen that was maintained for 12 days’ exposure to progressively increasing altitude. An additional 2 weeks’ acclimatization at 5050 m altitude rendered the pulmonary vasculature minimally responsive to oxygen similar to the fully acclimatized non‐ascent Sherpa. Additional hypoxic exposure at that time point did not augment hypoxic pulmonary vasoconstriction.

## 1 INTRODUCTION

The pulmonary vasculature in healthy humans at sea level is a low pressure, highly distensible system (Naeije & Chesler, 2012). The pulmonary vasculature constricts in response to alveolar hypoxia to maintain adequate ventilation–perfusion relationships and preserve gas exchange (Dorrington et al., 1997). During ascent to high altitude, the partial pressure of alveolar oxygen is progressively reduced throughout the lungs due to decreased barometric pressure leading to generalized hypoxic pulmonary vasoconstriction (HPV) (Dunham‐Snary et al., 2017; Motley et al., 1947; Moudgil et al., 2005; Swenson, 2013; Von Euler & Liljestrand, 1946).

In humans, HPV is variable, and vasoconstriction is further augmented by increased sympathetic activation that occurs with ascent to altitude, although hypocapnia mitigates the sympathetic activation (Hansen & Sander, 2003; Krasney, 1994; Scherrer et al., 2013; Simpson et al., 2020). When the hypoxic stimulus is brief (a few hours) HPV is completely reversible with restoration of normoxia or administration of 100% oxygen. As the duration of hypoxia increases, however, the reversibility of HPV to supplemental oxygen progressively decreases (Groves et al., 1987; Luks et al., 2017; Swenson, 2013; Sylvester et al., 2012), but the exact time course over which the HPV response goes from being acutely reversible to irreversible is not known (Frise & Robbins, 2015). This process occurs over a few days to several weeks and is believed to involve pulmonary vascular remodelling at the cellular level (Stenmark & McMurtry, 2005; Stenmark et al., 2015). Once this remodelling occurs, it is not known whether further – more severe – hypoxia will produce additional vasoconstriction although data from precapillary pulmonary hypertension patients suggests no augmentation of HPV (Carta et al., 2022; Swenson, 2013).

In a small study (*n* = 5) performed in acclimatized Tibetans at 3658 m, the administration of a hypoxic mixture (14% oxygen and 86% nitrogen) for 10 min did not significantly increase their mean pulmonary artery pressure (mPAP) or pulmonary vascular resistance (PVR) (Groves et al., 1993). On the other hand, a group of 13‐ to 17‐year‐old long‐term residents in Leadville, CO, USA (3094 m) demonstrated increased mPAP and PVR while breathing a mixture of 13% oxygen (Vogel et al., 1962). Once pulmonary vascular remodelling is present, it is not known how quickly the pulmonary vasculature regains the ability to ‘respond’ to supplemental oxygen with a lowering of pulmonary artery systolic pressure (PASP) and PVR in the presence of more oxygen.

In the echocardiographic study by Faoro and colleagues, PASP and total PVR (mPAP/cardiac output) at rest in Sherpa residents at high altitude was lower than in acclimatized lowlanders (Faoro et al., 2014). In Sherpa who spend a few days to a couple of weeks at low altitude before re‐ascent to high altitude, it is unclear how the pulmonary vasculature will respond.

To address these questions, we sought to determine the time course over which the pulmonary vascular response to hypobaric hypoxia changes from being fully reversible to potentially irreversible with supplemental oxygen by measuring serial changes in PASP and PVR in lowlanders and Sherpa during gradual ascent to 5050 m altitude. To ascertain the role of chronic hypoxia independent of the ascent, we also examined Sherpa who had not recently descended and remained at high altitude. We hypothesized that pulmonary vascular pressure in Sherpa during altitude ascent would demonstrate a blunted rise and would be less responsive to supplemental oxygen compared to lowlanders whereas Sherpa who did not descend would have minimal response to either supplemental O_2_ or to more severe inspired hypoxia.

## 2 METHODS

### 2.1 Ethical approval

Ethical approval was obtained from the research ethics committees at the University of British Columbia, Okanagan, Kelowna, BC, Canada (H16‐01028) and the Nepal Health Research Council. The studies conformed to the *Declaration of Helsinki*, apart from registration in a database. Lowlanders gave written informed consent in English prior to any study procedures. A Nepalese physician explained the study to Sherpa participants and these subjects signed an informed consent document written in Nepalese before any study procedures.

### 2.2 Participants

We recruited healthy adult volunteers (>18 years of age) in three different study groups: lowlanders and two groups of Sherpa. Additional details of the ascent profile and Sherpa study subjects have been published elsewhere (Willie et al., 2021, 2018). Although parts of this study have been previously published, the study aims and hypotheses in this paper were determined a priori with no overlap apart from the participants. Lowlanders (*n* = 20) were recruited from the University of British Columbia – Nepal research expedition. Adult Sherpa (>18 years of age) were born, raised and resided in the Khumbu area of Nepal at altitudes ≥3500 m. The first group of Sherpa (ascent Sherpa, *n* = 11) descended to Kathmandu (∼1300 m) for baseline testing and stayed in Kathmandu for 5–15 days (median 7 days) then re‐ascended with the expedition while participating in the testing sessions at the intermediate altitudes. A second group of Sherpa remained at high altitude (non‐ascent Sherpa, *n* = 12) without descent to below 3500 m prior to testing. The non‐ascent Sherpa travelled from their high‐altitude residence to the EV‐K2 CNR Pyramid Laboratory at 5050 m (near Lobuche) for testing. All the participants were healthy with no cardiac or respiratory medical problems. Individuals were screened for measurable tricuspid regurgitation before subsequent study procedures.

### 2.3 Demographics

All volunteers were males except one female lowlander. There was no significant age difference between the ascent Sherpa (*n* = 11, 35 ± 12 years old) (mean ± SD) and lowlanders (*n* = 20, 30 ± 8 years) but the non‐ascent Sherpa (*n* = 12, 23 ± 6 years) were slightly younger than the ascent Sherpa (*P* = 0.01) and lowlanders (*P* = 0.02). Body mass index was similar in lowlanders (23.4 ± 2.0 kg/m^2^) and the ascent Sherpa (23.3 ± 4.0 kg/m^2^, *P* = 0.97), but higher in lowlanders than the non‐ascent Sherpa (21.1 ± 1.4 kg/m^2^, *P* = 0.003).

### 2.4 Study protocol

For the lowlanders and the ascent Sherpa group, baseline measurements were performed in Kathmandu (1300 m). For the non‐ascent Sherpa group, all testing was performed in the Pyramid Laboratory (5050 m).

**FIGURE 1:**
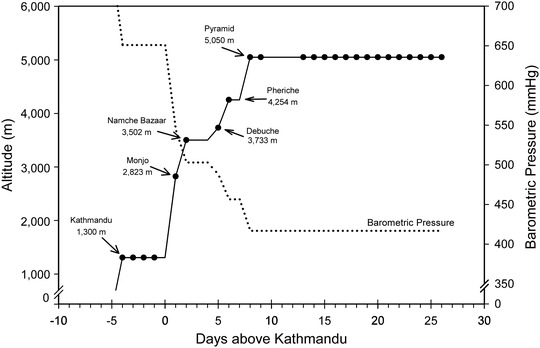
Ascent profile for the expedition showing testing sites and times by the filled circles on the continuous ascent profile line. Also shown is the barometric pressure (dotted line) during the ascent. Filled circles denote times and altitudes where echocardiographic measurements were obtained

After baseline testing the lowlanders and ascent Sherpa groups flew to Lukla (2860 m) and trekked from there to the Pyramid Laboratory over the next 8–10 days. The same protocol was applied to both lowlanders and ascent Sherpa groups at the following study points: Monjo (2823 m), Namche Bazaar (3502 m), Debuche (3733 m), Pheriche (4254 m) and Pyramid Laboratory (5050 m) (Figure 1). After 1–2 h rest at each new altitude, echocardiographic measurements were performed while breathing ambient air and then all subjects were placed on supplemental oxygen via facial mask to keep their $S_{pO_{2}}$ ≥ 96% for 30 min. Repeat echocardiograms were performed after 30 min while still breathing supplemental oxygen. All the participants ascended slowly so that there was no use of prophylactic medications such as acetazolamide for the treatment of high‐altitude illnesses.

For the non‐ascent Sherpa group, baseline echocardiograms were performed in the Pyramid Laboratory while breathing ambient air. After completion of baseline testing, they breathed supplemental oxygen via a facial mask to maintain their oxygen saturation ($S_{pO_{2}}$ ≥ 96%) for 30 min. A repeat echocardiogram was performed after 30 min while still breathing supplemental oxygen.

Late‐Pyramid testing was performed after subjects spent about 2 weeks continuously at 5050 m. For all groups (lowlanders, ascent Sherpa and non‐ascent Sherpa) PASP and PVR were measured breathing ambient air and supplemental oxygen. After completion of the supplemental oxygen testing, approximately 15–30 min was spent breathing ambient air before hypoxic testing commenced. Hypoxia was induced by breathing a gas mixture of ∼16% oxygen with balance nitrogen, $P_{IO_{2}}$ ∼67 mmHg via a facial mask for 15 min to simulate a further ascent of ∼2000 m during which a repeat echocardiogram was performed.

For purposes of this report pulmonary vascular ‘response’ to supplemental oxygen (or to increased hypoxia) is defined as a significant change in PVR, accompanied by a change in PASP or mPAP. An isolated change in pressure without an accompanying change in resistance was not considered a ‘response’ to oxygen or additional hypoxia since this would be a result of changes in cardiac output.

### 2.5 Experimental measures

Echocardiogram (Vivid Q, GE, Fairfield, CT, USA) images were obtained while subjects breathed ambient air and again while breathing supplemental oxygen. All echocardiographic images were obtained by one of two experienced investigators (M.S. or A.W.). Images were stored for subsequent analysis off‐line by the same investigators blinded to the study conditions. A left ventricular outflow tract Doppler tracing was performed and the velocity–time integral (VTI) was recorded. Stroke volume was determined from the Doppler signal using the VTI and the aortic cross section area (π × aortic diameter^2^ × 4^−1^) obtained from a parasternal long axis view. Cardiac output (CO) was calculated as the product of stroke volume and heart rate. PASP was measured by Doppler echocardiography based upon the measurement of the maximum velocity of the tricuspid regurgitation jet (Bertini et al., 2009). The peak systolic pressure gradient of the right ventricle (∆*P*_max_) to the right atrium was calculated according to the simplified Bernoulli equation (4 × *V* ^2^) without correcting for changes in haematocrit, where *V* is the peak systolic velocity of the tricuspid regurgitate. PASP was then determined by adding the right atrial pressure. Right atrial pressure was estimated by evaluation of inferior vena cava diameter and response to a deep inspiration (Aessopos et al., 2000). If inferior vena cava diameter was reduced by at least 50% during a deep inspiration, then a right atrial pressure of 5 mmHg was assumed. This approach has been used previously (Bossone & Armstrong, 1999) and is supported by right heart catheterization studies in healthy humans (Lang et al., 2015). No subjects were excluded because of an elevated right atrial pressure as all subjects had at least a 50% reduction in inferior vena cava diameter during a deep inspiration. PVR was calculated using Doppler signal from tricuspid regurgitation (TR) velocity and right ventricular outflow tract velocity time integral (RVOT‐VTI) where PVR was 10 × (TR velocity/RVOT‐VTI) + 0.16 (Rudski et al., 2010). Oxygen saturation ($S_{pO_{2}}$) was measured using a fingertip pulse oximeter after ensuring an adequate pulse waveform (Masimo Rad‐57^TM^, Masimo Corp., Irvine, CA, USA or similar device).

### 2.6 Statistical analysis

To assess the pulmonary vascular responsiveness to oxygen in lowlanders and Sherpa, data were analysed using a linear mixed‐effects model with a compound symmetry repeated measure co‐variance structure. Our primary outcome variables included PASP and PVR along with the change in these variables while breathing supplemental oxygen or a hypoxic gas mixture. The fixed factors in the model were ethnicity (Sherpa vs. lowlanders), oxygen (ambient air, supplemental O_2_, or additional hypoxia) and altitude with altitude being the repeated factor. Subjects were included in the model as a random effect. We anticipated that during time at the Pyramid Laboratory, subjects would develop less pulmonary vascular reactivity. As such the ‘late’ Pyramid Laboratory data were analysed separately, again using a linear mixed model with fixed and random effects as above. For both the ascent data and the late Pyramid data, *post hoc* tests using Student's paired *t* test were performed as appropriate when the overall response was significant. Statistical analyses were performed using SPSS v24 (SPSS Statistics, IBM Corp., Armonk, NY, USA) and R version 3.6.1 (R Foundation for Statistical Computing, Vienna, Austria). Results are reported as means ± SD and statistical significance was set at *P* < 0.05.

## 3 RESULTS

### 3.1 Pulmonary vascular changes during ascent

**FIGURE 2:**
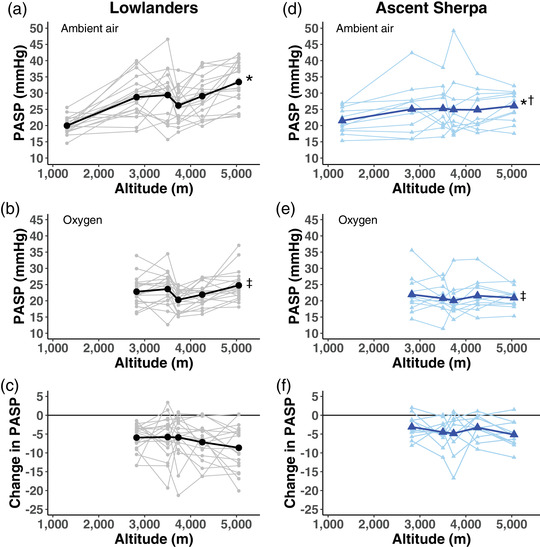
Time course of changes in PASP across altitude among lowlanders and ascent Sherpa. Larger bold symbols represent the mean values with the individual data points in lighter symbols. All values are in mmHg. (a) Ambient air PASP in lowlanders. (b) PASP breathing supplemental oxygen in lowlanders. (c) Change in PASP from ambient air to supplemental oxygen in lowlanders. (d) Ambient air PASP in Ascent Sherpa. (e) PASP breathing supplemental oxygen in ascent Sherpa. (f) Change in PASP from ambient air to supplemental oxygen in ascent Sherpa. *Significant increase in PASP with increasing altitude, *P* < 0.001. †Significant interaction between altitude and ancestry, *P* < 0.003. ‡Significant reduction in PASP following oxygen administration, *P* < 0.001. PASP, pulmonary artery systolic pressure

**FIGURE 3:**
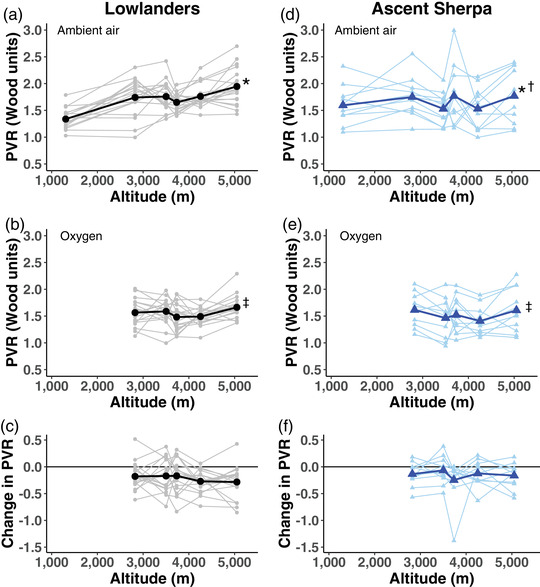
Time course of changes in PVR across altitude among lowlanders and ascent Sherpa. Larger bold symbols represent the mean values with the individual data points in lighter symbols. (a) Ambient air PVR in lowlanders. (b) PVR breathing supplemental oxygen in lowlanders. (c) Change in PVR from ambient air to supplemental oxygen in lowlanders. (d) Ambient air PVR in ascent Sherpa. (e) PVR breathing supplemental oxygen in ascent Sherpa. (f) Change in PVR from ambient air to supplemental oxygen in ascent Sherpa. *Significant increase in PVR with increasing altitude, *P* < 0.001. †Significant interaction between altitude and ancestry, *P* < 0.001. ‡Significant reduction in PVR following oxygen administration, *P* < 0.001. PVR, pulmonary vascular resistance

Lowlanders and ascent Sherpa trekked from low altitude to the Pyramid Laboratory at 5050 m. With this increase in altitude, PASP increased progressively in both ascent Sherpa and lowlanders (Table 1). Both lowlanders and ascent Sherpa had a significant decrease in PASP during oxygen administration. Across all altitudes, however, lowlanders had a trend toward a larger reduction in PASP following oxygen administration than did ascent Sherpa (*P* = 0.056, Figure 2 and Table 1). Like PASP, the PVR increased progressively with increasing altitude in ascent Sherpa and lowlanders. Although both groups had significant reductions in PVR with supplemental oxygen, these reductions were similar in both ethnic groups (*P* = 0.314, Figure 3 and Table 1).

**TABLE 1:** Changes in haemodynamics, cardiac output and other parameters across increasing altitudes

		KTM 1300 m	Monjo 2823 m	Namche 3502 m	Debouche 3733 m	Pheriche 4254 m	PYR‐arrival 5050 m	Altitude	Ancestry	Inter
PASP	Lowlander	20 ± 2.5 (19)	28.8 ± 6.3* (19)	29.4 ± 7.5* (19)	26.2 ± 5.1* (19)	29.1 ± 6.2* (19)	33.4 ± 6.5* (19)	**<0.001**	0.099	**0.003**
	Ascent Sherpa	21.5 ± 4 (11)	25 ± 7.8 (11)	25.2 ± 6.4 (11)	24.9 ± 9.5 (11)	24.8 ± 6 (11)	26.1 ± 4.9** (11)			
PVR	Lowlander	1.34 ± 0.18 (19)	1.74 ± 0.33* (19)	1.76 ± 0.28* (19)	1.65 ± 0.16* (19)	1.76 ± 0.22* (19)	1.95 ± 0.34* (19)	**<0.001**	0.643	**0.001**
	Ascent Sherpa	1.6 ± 0.35** (11)	1.75 ± 0.37 (11)	1.53 ± 0.3 (11)	1.77 ± 0.56 (11)	1.53 ± 0.39 (11)	1.77 ± 0.5 (11)			
$S_{pO_{2}}$	Lowlander	94 ± 2 (19)	92 ± 2 (18)	89 ± 7 (19)	88 ± 3 (19)	85 ± 4 (19)	80 ± 3 (19)	**<0.001**	0.086	0.254
	Ascent Sherpa	97 ± 2 (11)	94 ± 2 (10)	92 ± 2 (11)	88 ± 4 (11)	87 ± 3 (11)	81 ± 2 (11)			
HR	Lowlander	58 ± 11 (19)	72 ± 18* (19)	78 ± 17* (19)	72 ± 17* (19)	77 ± 17* (19)	75 ± 16* (19)	**<0.001**	0.789	**0.038**
	Ascent Sherpa	61 ± 10 (11)	65 ± 9 (11)	70 ± 13 (11)	71 ± 8 (11)	77 ± 10* (11)	81 ± 10* (11)			
CO	Lowlander	3.7 ± 0.8 (19)	4.1 ± 1.4 (19)	4.8 ± 1.7 (19)	4.1 ± 1.4 (19)	4.7 ± 1.5 (19)	4.4 ± 1.2 (19)	**<0.001**	**0.041**	0.326
	Ascent Sherpa	2.9 ± 0.7 (11)	3.2 ± 1 (11)	3.6 ± 1 (11)	3.2 ± 0.7 (11)	3.9 ± 0.8 (11)	4 ± 1.2 (11)			
ΔPASP	Lowlander		5.9 ± 3.6 (19)	5.8 ± 5.5 (19)	5.9 ± 5.5 (19)	7.2 ± 5.1 (19)	8.6 ± 6 (19)	0.201	0.056	0.533
	Ascent Sherpa		3.1 ± 3.2 (11)	4.5 ± 3.4 (11)	4.8 ± 5.3 (11)	3.3 ± 3.2 (11)	5.2 ± 3.5 (11)			
ΔPVR	Lowlander		0.18 ± 0.24 (19)	0.17 ± 0.3 (19)	0.17 ± 0.31 (19)	0.27 ± 0.2 (19)	0.28 ± 0.3 (19)	0.456	0.314	0.402
	Ascent Sherpa		0.13 ± 0.22 (11)	0.07 ± 0.25 (11)	0.25 ± 0.39 (11)	0.12 ± 0.24 (11)	0.16 ± 0.24 (11)			

Data are means ± SD (*n*), where *n* is number of subjects. Linear mixed effects model with a compound symmetry repeated measure co‐variance structure. The fixed factors for the model were altitude (i.e., location) and ancestry (i.e., group), with altitude being a repeated factor. Subjects were included as a random effect. When a significant interaction effect (e.g., altitude × ancestry) was detected, Bonferroni adjusted *post hoc* tests were utilized to test pairwise comparisons. Values in bold indicate statistical significant with values less than 0.05.

* *P* < 0.05 compared to KTM.

** *P* < 0.05 compared to lowlanders at that altitude/time. Abbreviations: CO, cardiac output, l/min; ΔPASP, change in PASP from ambient air to supplemental O_2_; ΔPVR, change in PVR from ambient air to supplemental O_2_; HR, heart rate, beats per minute; Inter, significant interaction; KTM, Kathmandu; PASP, pulmonary artery systolic pressure, mmHg; PVR, pulmonary vascular resistance, Wood units; $S_{pO_{2}}$, pulse oximetry oxygen saturation, %.

### 3.2 Pulmonary vascular responses to supplemental O_2_ during prolonged stay at 5050 m

**FIGURE 4:**
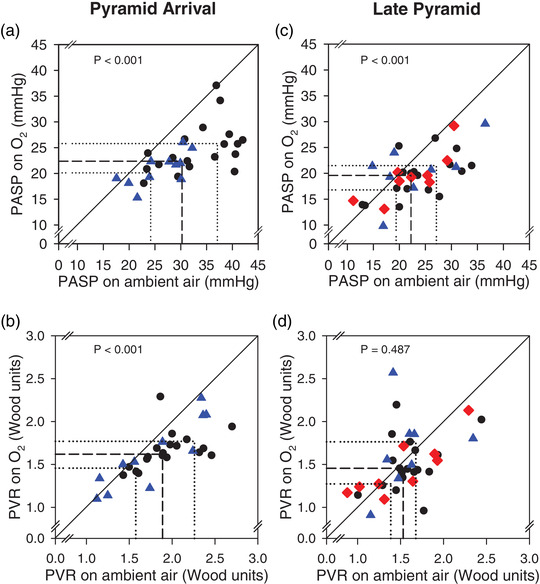
Individual data for PASP and PVR on ambient air and on supplemental oxygen on arrival to the Pyramid Laboratory (a, b) and after ∼2 weeks additional acclimatization at 5050 m (c, d). Circles are lowlanders, triangles are ascent Sherpa, and diamonds are non‐ascent Sherpa. Continuous diagonal lines show the line of identity (no change with oxygen). Dashed lines show the median values with the thin dotted lines showing the 25th percentile and 75th percentile. *P*‐values reflect the paired comparison on and off supplemental oxygen

**FIGURE 5:**
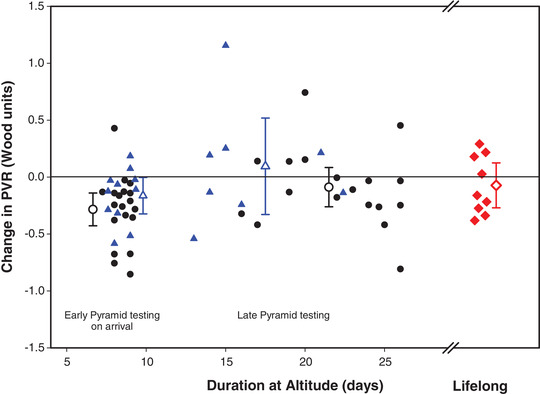
Change in PVR from breathing ambient air to supplemental oxygen plotted as a function of time at altitude. Duration at altitude reflects the time each subject spent at altitude above Kathmandu. Filled symbols represent the individual subject data while the open symbols with error bars represent the mean change ±95% CI for the PVR change with oxygen. Circles represent the lowlanders, triangles are the ascent Sherpa, and the diamonds are the non‐ascent Sherpa. A few of the data points have been shifted slightly along the *x*‐axis to avoid overlapping symbols. Note, values less than zero represent a decrease in PVR while breathing supplemental oxygen

Lowlanders and ascent Sherpa were tested immediately after arrival to the Pyramid Laboratory (early Pyramid testing). After an average of 14 days further stay at the Pyramid Laboratory (5050 m) these individuals were re‐tested (late Pyramid testing) along with the non‐ascent Sherpa group. In this late Pyramid testing, although PASP decreased with supplemental oxygen in all three groups combined (*P* < 0.001, Figure 4), PVR was unchanged (*P* = 0.49; Table 2 and Figure 5).

**TABLE 2:** Effects of oxygen inhalation on PASP, PVR, cardiac output and $S_{pO_{2}}$ at various altitudes

			Ambient Air	O_2_	Hypoxia	Ancestry	O_2_	Inter
Monjo 2823 m	PASP	Lowlanders	28.8 ± 6.3 (19)	22.8 ± 4.5 (19)*	—	0.298	**<0.001**	**0.042**
		Ascent Sherpa	25.0 ± 7.8 (11)	21.9 ± 5.8 (11)*	—			
	PVR	Lowlanders	1.74 ± 0.33 (19)	1.56 ± 0.25 (19)	—	0.789	**0.002**	0.631
		Ascent Sherpa	1.75 ± 0.37 (11)	1.61 ± 0.30 (11)	—			
	CO	Lowlanders	4.13 ± 1.36 (19)	3.49 ± 0.86 (19)	—	**0.008**	**0.003**	0.821
		Ascent Sherpa	3.24 ± 0.98 (11)	2.50 ± 0.51 (11)	—			
	$S_{pO_{2}}$	Lowlanders	91.7 ± 2.30 (18)	98.9 ± 0.90 (19)*	—	**0.031**	**<0.001**	**0.008**
		Ascent Sherpa	94.1 ± 2.4 (10)^†^	98.8 ± 0.8 (11)^†^	—			
Namche 3502 m	PASP	Lowlanders	29.4 ± 7.5 (19)	23.6 ± 5.1 (19)	—	0.112	**<0.001**	0.485
		Ascent Sherpa	25.2 ± 6.4 (11)	20.8 ± 4.6 (11)	—			
	PVR	Lowlanders	1.76 ± 0.28 (19)	1.59 ± 0.24 (19)	—	0.071	**0.035**	0.33
		Ascent Sherpa	1.53 ± 0.30 (11)	1.46 ± 0.36 (11)	—			
	CO	Lowlanders	4.79 ± 1.68 (19)	3.85 ± 1.39 (19)	—	**0.041**	**<0.001**	0.236
		Ascent Sherpa	3.55 ± 0.99 (11)	3.00 ± 0.78 (11)	—			
	$S_{pO_{2}}$	Lowlanders	89.1 ± 7.1 (19)	99.7 ± 0.6 (19)	—	0.234	**<0.001**	0.189
		Ascent Sherpa	91.9 ± 2.2 (11)	99.6 ± 0.5 (11)	—			
Debuche 3733 m	PASP	Lowlanders	26.2 ± 5.1 (19)	20.3 ± 3.2 (19)	—	0.689	**<0.001**	0.608
		Ascent Sherpa	24.9 ± 9.5 (11)	20.1 ± 4.9 (11)	—			
	PVR	Lowlanders	1.65 ± 0.16 (19)	1.48 ± 0.26 (19)	—	0.455	**0.003**	0.55
		Ascent Sherpa	1.77 ± 0.56 (11)	1.52 ± 0.34 (11)	—			
	CO	Lowlanders	4.08 ± 1.36 (19)	3.15 ± 0.98 (19)	—	0.054	**<0.001**	0.388
		Ascent Sherpa	3.23 ± 0.69 (11)	2.52 ± 0.65 (11)	—			
	$S_{pO_{2}}$	Lowlanders	88.5 ± 3.3 (19)	99.0 ± 0.7 (19)	—	0.377	**<0.001**	0.734
		Ascent Sherpa	87.6 ± 3.8 (11)	98.6 ± 0.8 (11)	—			
Pheriche 4254 m	PASP	Lowlanders	29.1 ± 6.2 (19)	21.9 ± 3.5 (19)	—	0.21	**<0.001**	**0.029**
		Ascent Sherpa	24.8 ± 6.0 (11)^†^	21.6 ± 5. (11)	—			
	PVR	Lowlanders	1.76 ± 0.22 (19)	1.49 ± 0.16 (19)	—	0.077	**<0.001**	0.082
		Ascent Sherpa	1.53 ± 0.39 (11)	1.41 ± 0.26 (11)	—			
	CO	Lowlanders	4.70 ± 1.45 (19)	3.52 ± 1.05 (19)	—	0.102	**<0.001**	0.191
		Ascent Sherpa	3.86 ± 0.84 (11)	3.02 ± 0.57 (11)	—			
	$S_{pO_{2}}$	Lowlanders	84.9 ± 4.4 (19)	98.9 ± 0.8 (19)^†^	—	0.299	**<0.001**	**0.039**
		Ascent Sherpa	87.5 ± 3.4 (11)	98.1 ± 0.3 (11)*	—			
Pyr‐Arr 5050 m	PASP	Lowlanders	33.4 ± 6.5 (19)	24.8 ± 4.8 (19)	—	**0.003**	**<0.001**	0.09
		Ascent Sherpa	26.1 ± 4.9 (11)	20.9 ± 3.1 (11)	—			
	PVR	Lowlanders	1.95 ± 0.34 (19)	1.66 ± 0.21 (19)	—	0.358	**<0.001**	0.263
		Ascent Sherpa	1.77 ± 0.50 (11)	1.61 ± 0.40 (11)	—			
	CO	Lowlanders	4.38 ± 1.15 (19)	3.60 ± 1.03 (19)	—	0.185	**<0.001**	0.207
		Ascent Sherpa	4.04 ± 1.21 (11)	2.89 ± 0.87 (11)	—			
	$S_{pO_{2}}$	Lowlanders	79.8 ± 3.3 (19)	98.5 ± 0.7 (17)	—	0.329	**<0.001**	0.245
		Ascent Sherpa	81.1 ± 2.3 (11)	98.4 ± 0.7 (11)	—			
Pyr‐Late 5050 m	PASP	Lowlanders	23.7 ± 5.8 (18)	18.7 ± 4.7 (18)	24.1 ± 9.0 (17)	0.467	**<0.001**	0.355
		Ascent Sherpa	23.1 ± 7.5 (8)	20.4 ± 5.6 (8)	29.1 ± 8.1 (8)			
		Non‐ascent Sherpa	21.2 ± 6.3 (12)	19.5 ± 4.6 (9)	23.5 ± 6.6 (9)			
	PVR	Lowlanders	1.60 ± 0.30 (18)	1.51 ± 0.31 (18)	1.56 ± 0.46 (17)	0.613	0.487	0.749
		Ascent Sherpa	1.58 ± 0.35 (8)	1.67 ± 0.48 (8)	1.69 ± 0.28 (8)			
		Non‐ascent Sherpa	1.49 ± 0.43 (12)	1.46 ± 0.33 (9)	1.61 ± 0.34 (9)			
	CO	Lowlanders	4.60± 0.88 (18)	3.91 ± 0.64 (18)	5.23 ± 1.06 (17)	0.373	**<0.001**	0.693
		Ascent Sherpa	4.82 ± 1.27 (8)	4.18 ± 1.44 (8)	5.49 ± 0.73 (8)			
		Non‐ascent Sherpa	4.31 ± 1.04 (12)	3.37 ± 0.54 (11)	5.23 ± 1.42 (11)			
	$S_{pO_{2}}$	Lowlanders	87.3 ± 2.6 (18)	98.5 ± 0.8 (18)	74.8 ± 6.6 (17)	**0.002**	**<0.001**	**0.002**
		Ascent Sherpa	84.1 ± 2.2 (8)	97.9 ± 0.6 (8)	62.7 ± 3.5 (8)			
		Non‐ascent Sherpa	87.1 ± 3.5 (12)	98.5 ± 0.8 (12)	71.0 ± 11.5 (12)			

Data are means ± SD (*n*), where *n* is number of subjects. Linear mixed effects model performed at each altitude. At the late Pyramid testing statistical comparisons are only between Lowlanders and the Ascent Sherpa. Values in bold indicate statistical significant with values less than 0.05. Abbreviations: CO, cardiac output, l/min; HR, heart rate, beats per minute; Inter, significant interaction; KTM, Kathmandu; PASP, pulmonary artery systolic pressure, mmHg; PVR, pulmonary vascular resistance, Wood units; Pyr‐Arr, Pyramid arrival testing; Pyr‐Late, Late Pyramid testing; $S_{pO_{2}}$, pulse oximetry oxygen saturation, %.

^†^ *p* < 0.05, lowlanders vs Ascent Sherpa.

*p < 0.05, Ambient Vs Supplemental Oxygen.

### 3.3 Pulmonary vascular changes in response to changes in $S_{pO_{2}}$

**FIGURE 6:**
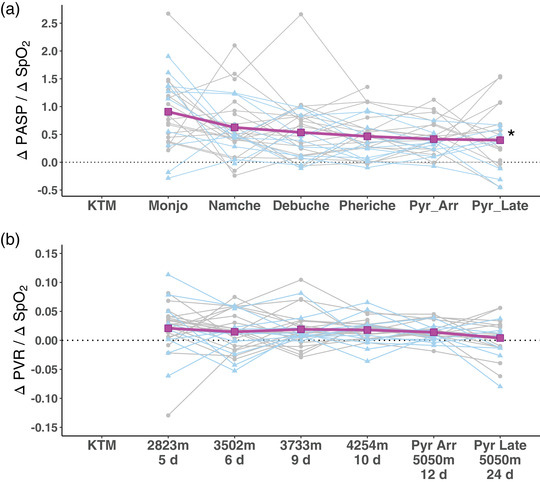
Changes in PASP and PVR (from ambient air to oxygen breathing) normalized to the accompanying changes in $S_{pO_{2}}$ (ambient air to oxygen breathing) at different altitudes. (a) Changes in PASP normalized to the accompanying changes in $S_{pO_{2}}$ (ΔPASP/Δ$S_{pO_{2}}$) at increasing altitudes. *P < 0.001 for altitude effect in the linear mixed model (LMM). There was no significant effect of ancestry (*P* = 0.255) or altitude by ancestry interaction (*P* = 0.885). Addition of cardiac output to the LMM did not improve the model fit. (b) Changes in PVR from ambient air to oxygen normalized to the accompanying changes in $S_{pO_{2}}$ (ΔPVR/Δ$S_{pO_{2}}$) at increasing altitude. LMM analysis showed no significant effects, altitude effect (*P* = 0.171), ancestry effect (*P* = 0.311) and interaction (*P* = 0.767). The addition of cardiac output to the LMM did not improve the fit of the model

At each altitude changes in PASP in response to a unit change in $S_{pO_{2}}$ after supplemental oxygen were assessed. ΔPASP/Δ$S_{pO_{2}}$ decreased from 0.9 ± 0.6 at 2823 m to 0.2 ± 0.6 at 5050 m (*P* < 0.001 by linear mixed model analysis); however there was no effect of ancestry (*P* = 0.255) or an altitude–ancestry interaction (*P* = 0.885) (Figure 6a). Similarly changes in PVR to a unit change in $S_{pO_{2}}$ after supplemental oxygen were calculated. The linear model showed no significant effect of altitude (*P* = 0.171), ancestry (*P* = 0.311) or interaction (*P* = 0.767) for PVR (Figure 6b).

### 3.4 PASP and PVR responses to additional hypoxia during prolonged stay at 5050 m

At the late Pyramid testing point, lowlanders, ascent Sherpa and non‐ascent Sherpa were studied while breathing a hypoxic gas mixture to simulate additional ascent of ∼2000 m. Due to time constraints, out of 43 volunteers in all three groups a total of 34 volunteers were studied (17 lowlanders, 8 ascent Sherpa, 9 non‐ascent Sherpa). With more severe hypoxia, PASP was unchanged (23.2 ± 6.2 mmHg on ambient air versus 25.1 ± 8.3 mmHg with hypoxia, with a mean change of 1.9 ± 7.1 mmHg (95% CI: −4.4 to 0.6 mmHg); *P* = 0.13). In these subjects PVR also remained unchanged with additional hypoxia. PVR on ambient air was 1.58 ± 0.35 Wood units and 1.60 ± 0.39 Wood units with hypoxia, with a mean change of 0.03 ± 0.38 Wood units (95% CI: −0.16 to 0.10 Wood units) (Figure 5).

## 4 DISCUSSION

Our study demonstrated two novel findings. (1) In both lowlanders and Sherpa who had spent 5–15 days at low altitude (ascent Sherpa), pulmonary vascular responsiveness to oxygen was maintained for 12 days’ exposure to progressively increasing altitude. After a further ∼14 days at 5050 m these individuals’ pulmonary vasculature became unresponsive to supplemental oxygen. (2) After ∼26 days at 5050 m, more severe hypoxia (or hyperoxia) did not augment HPV in lowlanders or either group of Sherpa. These latter findings indicate possible remodelling of the pulmonary vasculature after 3 weeks at 5050 m.

In lowlanders, our finding of an increase in PASP and PVR with ascent to higher altitude is consistent with the findings of others. HPV is initially reversible with 100% oxygen supplementation (as confirmed in our early Pyramid testing), but when the exposure is more prolonged, HPV becomes irreversible with supplemental oxygen. Hultgren et al. studied 21 healthy acclimatized adult males in Peru (3750 m and 4328 m) where 100% supplemental oxygen was administered for 10–15 min and invasive haemodynamic measurements were completed during the final 4 min (Hultgren et al., 1965). The mPAP significantly decreased but did not fall to normal sea‐level values. There was no change in PVR suggesting pulmonary vascular remodelling.

The regression of pulmonary pressures and PVR on return to sea level after prolonged residence at altitude is poorly understood but has been studied in a several pieces of work with 10–11 subjects with variable findings (Penaloza & Sime, 1971; Sime et al., 1971). Two years’ residence at sea level in high‐altitude Andeans caused normalization of pulmonary artery pressures and resistance at rest, but the pulmonary artery pressure response to exercise remained higher than normal sea level responses (Banchero & Cruz, 1970). Hartley et al. studied 10 long‐term residents of Leadville, CO, USA (3100 m) at altitude and after 10 days’ residence at sea level (Hartley et al., 1967). The descent to sea level did not alter pulmonary artery pressures or resistance. In contrast, Hilty et al. found that the elevated pulmonary artery pressure after 4 weeks of continuous exposure to high altitude in lowlanders reverts to normal when studied for 7 days following rapid descent (Hilty et al., 2016). To our knowledge, ours is the first study to evaluate these findings in acclimatized Sherpa, and our data indicate that 5–15 days at low altitude in Sherpa altered their pulmonary vasculature such that when re‐exposed to altitude it responds similar to that of unacclimatized lowlanders.

Our study allowed the direct comparison of Sherpa and lowlanders with similar ascent profiles at various points in time. As expected, PASP increased in both groups with increasing altitude. PVR also increased in both groups with increasing altitude, but lowlanders had a larger increase in PVR than Sherpa suggesting residual acclimatization or residual pulmonary vascular remodelling in the Sherpa. These findings are similar to the findings by Luks et al. who measured tricuspid valve transvalvular pressure gradient (TPVG) in 11 lowlanders (8 men and 3 women) in two locations, Namche Bazaar (3500 m) and Everest Base Camp (EBC, 5300 m) during gradual ascent to EBC (Luks et al., 2017). Measurements were performed breathing ambient air, hypoxic mixture and hyperoxia. They concluded that acute pulmonary vascular responsiveness to hypoxia does not change over the first 12–13 days of progressive high‐altitude exposure.

Tibetans are thought to be the ancestral population from which modern Sherpa have descended (Bhandari et al., 2015, 2017). As such, the pulmonary vascular responses should be similar in our Sherpa compared with Tibetans. In five normal 22‐year‐old Tibetan males studied by Groves et al. in Lhasa (3658 m), pulmonary artery pressure was normal while breathing ambient air and did not significantly change with hypoxia (Groves et al., 1993). After 10 min of breathing 100% oxygen, there was no change in pulmonary artery pressure. But contrary to those findings, our study showed that PASP decreased in the ascent Sherpa with supplemental oxygen similar to the findings of others in Sherpa (Foster et al., 2014) and acclimatized Andeans (Hultgren et al., 1965). Interestingly, though, after 14 days further stay at 5050 m (late Pyramid testing), lowlanders and both Sherpa groups did not show any increase in PASP or PVR to additional hypoxic stimulus; these findings are in contrast to those from long‐term but younger (13‐17 year old) residents in Leadville, CO (Vogel et al., 1962).

Echocardiographic measurements of PASP, PVR and cardiac output are connected. At each altitude studied there was a decrease in cardiac output during the supplemental oxygen condition (Table 2). This occurred both at the Pyramid arrival time and again a couple weeks later at the late Pyramid testing. With oxygen breathing at the late Pyramid testing, PVR did not decrease despite the decrease in cardiac output strongly suggesting pulmonary vascular changes likely are responsible for this finding.

### 4.1 Limitations

One of the limitations of our study was not being able to record PASP and PVR data with supplemental oxygen at Kathmandu (1300 m). It is possible that even 1300 m may have altered the pulmonary vasculature in the lowlanders and the ascent Sherpa. Due to time constraints and the need for baseline data collection for multiple studies as a part of the expedition, we were not able to collect supplemental oxygen data in Kathmandu. Due to testing schedule constraints, the acclimatization time for lowlanders and ascent Sherpa at the Pyramid Laboratory varied somewhat as shown in Figures 1 and 5. Nevertheless, during this late testing time period, none of the groups demonstrated HPV responsiveness to supplemental oxygen or to additional hypoxia. When assessing the change in pulmonary vasculature to oxygen, the effect of cardiac output and haematocrit could be the confounding factors. Our results did not significantly change when cardiac output was entered in the mixed effects model. Luks et al. did not find any significant change in tricuspid valve pressure gradient and $S_{pO_{2}}$ slope when adjusted for difference in haematocrit (Luks et al., 2017). Partial pressure of carbon dioxide in blood ($P_{\mathrm{aC}O_{2}}$) affects pulmonary vascular tone (Balanos et al., 2003; Swenson, 2013) but $P_{\mathrm{aC}O_{2}}$ was not measured in our subjects during these studies and is not accounted for in our model. However, during very comparable time points during ascent and over time at 5050 m, $P_{\mathrm{aC}O_{2}}$ was within 1–3 mmHg between our three groups studied (Willie et al., 2021), and it would seem unlikely that this would play a major role. The hypoxic ventilatory response is highly variable among different individuals and may have affected our results via its influence on alveolar $P_{O_{2}}$. Unfortunately, the hypoxic ventilatory response was not measured in this study.

HPV is largely altered by changes in alveolar $P_{O_{2}}$ and to a lesser extent by mixed venous $P_{O_{2}}$ with multiple additional factors modulating this response (Swenson, 2013; Sylvester et al., 2012). In this study, only $S_{pO_{2}}$ as a crude surrogate for alveolar $P_{O_{2}}$ was measured, likely accounting for the lack of change over time at altitude to the PVR response to supplemental oxygen as a function of change in peripheral oxygen saturation (ΔPVR/Δ$S_{pO_{2}}$).

Another limitation is that supplemental oxygen may have altered sympathetic tone or the production of reactive oxygen species by effectively ‘lowering’ the altitude below that at Kathmandu. The $S_{pO_{2}}$ values with supplemental oxygen during ascent were somewhat higher than the ambient air $S_{pO_{2}}$ in Kathmandu of 94 ± 2% and 97 ± 2% in the lowlanders and ascent Sherpa, respectively. This adverse influence would seem unlikely, however, since neither groups were hyperoxic. Finally, the acclimatization time for the Sherpa and lowlander participants varied by several days and this may have affected the individual results but is unlikely to have altered the major findings of this study.

## 5 CONCLUSIONS

The data show increasing pulmonary vascular pressures during ascent to high altitude in both lowlanders and ascent Sherpa. Both these groups show decrease in pulmonary vascular pressure and resistance to supplemental oxygen administration during this ascent. After spending 5–15 days at low altitude, pulmonary vascular responses of the ascent Sherpa following re‐ascent to altitude were similar to those of lowlanders. After a further 14‐day stay at 5050 m there was no change in pulmonary vascular responses to both supplemental oxygen and hypoxia in lowlanders and ascent Sherpa as also seen in the fully acclimatized non‐ascent Sherpa. Additional studies are needed to elicit the precise mechanisms underlying the timing of how HPV goes from being fully reversible to irreversible.

## AUTHOR CONTRIBUTIONS

Conception and design of the work: Prajan Subedi, Michael Stembridge, Philip N. Ainslie and James D. Anholm, acquisition: Prajan Subedi, Christopher Gasho, Michael Stembridge, Alexandra Williams, Philip N. Ainslie and James D. Anholm; analysis: Prajan Subedi, Christopher Gasho, Michael Stembridge, Alexander Patrician, Philip N. Ainslie and James D. Anholm, or interpretation of data for the work: Prajan Subedi, Christopher Gasho, Michael Stembridge, Alexandra M. Williams, Alexander Patrician, Philip N. Ainslie and James D. Anholm; and drafting of the work or revising it critically for important intellectual content: Prajan Subedi, Christopher Gasho, Michael Stembridge, Alexandra M. Williams, Alexander Patrician, Philip N. Ainslie and James D. Anholm. All authors have read and approved the final version of this manuscript and agree to be accountable for all aspects of the work in ensuring that questions related to the accuracy or integrity of any part of the work are appropriately investigated and resolved. All persons designated as authors qualify for authorship, and all those who qualify for authorship are listed.

## CONFLICT OF INTEREST

All authors declare that they have no competing interests or conflicts of interest to disclose.

## FUNDING INFORMATION

There was no external funding for this study.

## Supporting information

Statistical Summary Document

## Data Availability

The data that support the findings of this study are available from the corresponding author upon reasonable request.
